# Differential Associations Between Sleep Domains and Response to Prolonged Exposure Therapy

**DOI:** 10.3390/bs15121654

**Published:** 2025-12-02

**Authors:** David L. Yap, Brooklynn Bailey, Hanah B. Weldon, Daniel F. Gros, Ron Acierno, Wendy Muzzy, Melba A. Hernandez-Tejada

**Affiliations:** 1 The Graduate Center, City University of New York, New York, NY 10016, USA; 2 Department of Psychiatry & Behavioral Sciences, Medical University of South Carolina, Charleston, SC 29425, USA; 3 Mental Health Service, Ralph H. Johnson Veterans Affairs Healthcare System, Charleston, SC 29401, USA; 4 Faillace Department of Psychiatry, University of Texas Health Sciences Center Houston, Houston, TX 77030, USA

**Keywords:** trauma, sleep, prolonged exposure, veterans

## Abstract

Prolonged Exposure (PE) is an effective evidence-based psychotherapy for posttraumatic stress disorder (PTSD); however, a small subset of veterans fails to achieve meaningful symptom reduction from PE. Given sleep’s role in memory consolidation, poor sleep quality may adversely affect fear extinction learning central to PE. Existing research on sleep and PE response often relies on single-item or global measures that miss nuanced sleep processes, assesses sleep as static (e.g., at baseline) rather than as a dynamic process, or focuses on concurrent rather than prospective associations. This study used a multidimensional measure of sleep quality to evaluate whether changes across several domains of sleep concurrently and prospectively predicted reduced PTSD symptoms. Sleep quality was assessed pre- and post-treatment and PTSD symptoms were measured pre- and post-treatment and at 3- and 6-month follow-ups. Changes in sleep domains were analyzed as predictors of concurrent and prospective PTSD symptoms. Improvements in overall sleep quality, subjective sleep quality, and daytime dysfunction were associated with improvements in PTSD symptom severity from pre- to post-treatment. Greater improvements in overall sleep quality and daytime dysfunction predicted lower PTSD severity at follow-up; however, greater reductions in daytime dysfunction predicted symptom increases across follow-up. Our findings highlight the importance of overall sleep quality in PE response and suggest that daytime dysfunction due to sleep problems may contribute to diminished long-term outcomes. Targeting these aspects of sleep may enhance treatment efficacy.

## 1. Sleep Quality Concurrently and Prospectively Predicts Response to Prolonged Exposure

Exposure-based therapies are widely recognized as first-line treatments for posttraumatic stress disorder (PTSD; VA/DoD, 2023), yet many individuals continue to experience significant symptoms following treatment. Clinical trials indicate that a substantial proportion of individuals—ranging from 20% to over 60%—retain a PTSD diagnosis after completing trauma-focused therapies such as Prolonged Exposure (PE) and Cognitive Processing Therapy (CPT) (Galovski et al., 2016; López et al., 2017; Steenkamp et al., 2015; Taylor et al., 2020). This variability in response highlights the need to identify factors that may interfere with treatment effectiveness.

One such factor is poor sleep quality, which is highly prevalent among individuals with PTSD (Caldwell et al., 2019; Germain et al., 2005; Troxel et al., 2015) and is believed to disrupt key mechanisms of change in exposure-based therapies, including fear extinction, memory consolidation, and emotional regulation (Pace-Schott et al., 2009; Walker & van der Helm, 2009). Poor sleep quality may be particularly detrimental to PE (Hunt et al., 2023), a gold-standard, manualized treatment that targets trauma-related avoidance through imaginal and in vivo exposures to fear stimuli (Foa et al., 2007). According to emotional processing theory, the therapeutic benefit of PE stems from activating trauma memories in a safe context to facilitate fear extinction and modify maladaptive beliefs (Foa & Kozak, 1986). These learning processes depend on intact memory consolidation and emotional regulation—functions that are closely tied to sleep, particularly REM and slow-wave sleep (Walker & Stickgold, 2004; Walker & van der Helm, 2009). Disrupted or insufficient sleep may impair these processes, reducing individuals’ capacity to benefit from exposure-based learning. Indeed, animal and human research shows that sleep loss impairs extinction retention and safety signal learning (Spoormaker et al., 2010; Straus et al., 2015), which are core mechanisms thought to underlie the effectiveness of PE.

Despite a growing body of research evaluating sleep quality as a factor influencing trauma-focused therapy outcomes, findings remain inconsistent. Some studies show that poor sleep—either before or during treatment—is associated with attenuated symptom improvement. For example, Zayfert and DeViva (2004) found that improvements in sleep over the course of treatment were associated with greater reductions in PTSD symptoms, and López et al. (2017) reported that residual sleep problems following treatment predicted poorer long-term outcomes. In a large randomized clinical trial, Taylor et al. (2020) observed that clinically significant insomnia, nightmares, and probable sleep apnea at baseline were linked to higher PTSD symptoms throughout treatment. However, all three of these studies relied on single-item or brief, static assessments of sleep, which may have limited their ability to capture meaningful individual differences or changes in sleep quality. Several investigations into the role of sleep in response to PTSD treatment have used the Pittsburgh Sleep Quality Index (PSQI; Buysse et al., 1989), which assesses seven domains of sleep quality (e.g., sleep efficiency, total sleep time) as well as global sleep quality. However, their findings have also been mixed. Hunt et al. (2023) found that change in sleep efficiency—but not total sleep time—predicted reductions in PTSD symptoms during PE, although their analyses included only two of the seven PSQI component scores. In contrast, Sexton et al. (2017) analyzed both global and component PSQI scores at baseline and found that sleep latency and sleep medication use were predictive of outcomes, whereas the global score and most other components were not. Park et al. (2025) only used the PSQI global score at baseline and found no moderating effect on symptom change across CPT.

These inconsistencies may reflect differences in study design and assessment of sleep. Sleep quality is a multifactorial construct that spans multiple domains, including duration, continuity, timing, and regularity (Buysse, 2014; Germain, 2013). Individuals with PTSD often exhibit short and irregular sleep, delayed bed and wake times, and fragmented sleep with low efficiency (Rezaie et al., 2018; Colvonen et al., 2019). These patterns may reflect distinct sleep phenotypes that could have differential associations with emotional dysregulation, trauma processing, and treatment response. Reliance on global indices or single sleep items may fail to capture which aspects of sleep are most relevant for clinical improvement. A more nuanced understanding of sleep quality—one that captures its multidimensional structure—may help clarify inconsistent findings in the literature and identify specific sleep patterns that interfere with recovery.

Furthermore, most studies examining the role of sleep in PTSD treatment have focused on baseline sleep quality, which can potentially inform treatment planning. In addition to baseline sleep, within-person changes across treatment may provide insights as to the relationship between sleep and response to PE. Sleep is a dynamic process that can vary substantially within individuals over time. Evidence suggests that within-treatment changes in sleep may offer important insights into recovery processes. For example, Zayfert and DeViva (2004) found that symptom improvement was strongest among patients whose sleep also improved over the course of cognitive behavioral treatment. Similarly, Hunt et al. (2023) found that veterans whose sleep efficiency, but not total sleep time, improved during PE evidenced greater fear extinction. In contrast, Gutner et al. (2013) reported that although PTSD symptoms decreased during cognitive processing therapy, insomnia symptoms persisted or even worsened in a subset of patients. Similarly, Pruiksma et al. (2023) found that veterans with poorer pre-treatment sleep quality experienced worsening of nightmares and insomnia during PE, which was associated with poorer PTSD outcomes. Importantly, none of these studies evaluated how sleep changes *during* treatment predicted PTSD symptoms at *follow-ups* to treatment. These findings underscore the need for research that uses repeated, multidimensional assessments of sleep as well as follow-up assessments of PTSD symptoms to better understand sleep’s role in treatment response.

The present study examined the relationship between sleep quality and PTSD symptom change in U.S. military veterans enrolled in a large randomized control trial investigating peer support augmentation of PE for PTSD (Hernandez-Tejada et al., 2024). Sleep quality was assessed multidimensionally at baseline and posttreatment. PTSD symptoms were assessed at baseline, posttreatment, and follow-up. Although the global sleep quality score aggregates across distinct sleep domains, we included it to allow for direct comparison with prior work and help situate our multidimensional approach within the existing literature. We tested whether changes in sleep quality across treatment predicted changes in PTSD symptoms both concurrently and prospectively at follow-up. Given the mechanistic role of memory consolidation during sleep (Walker & Stickgold, 2004)—particularly its relevance to fear extinction (Spoormaker et al., 2010; Straus et al., 2015; Walker & van der Helm, 2009)—as well as prior findings linking improvement in sleep efficiency to stronger treatment response (Hunt et al., 2023), we expected that changes in the sleep indices most proximal to memory consolidation would have the strongest relationships with PTSD symptom improvement. Thus, we hypothesized that greater reductions in sleep disturbance, along with greater increases in sleep duration, sleep efficiency, and global sleep quality, would be most strongly associated with improvements in PTSD symptoms at posttreatment and follow-up. In contrast, we did not expect subjective sleep quality, sleep latency, sleep medication use, or daytime dysfunction to significantly predict treatment outcomes, as these domains are more distally related to the sleep-dependent consolidation processes thought to support exposure-based learning (Walker & van der Helm, 2009).

## 2. Methods

### 2.1. Participants

The study sample consisted of 109 U.S. military veterans recruited from a Veterans Affairs Healthcare System (VAHCS) in the southeastern United States. Participants in the current study were part of a larger randomized control trial investigating peer support augmentation of PE for PTSD (Hernandez-Tejada et al., 2024). All participants had previously initiated, but had failed to complete an evidence-based, exposure-based treatment for PTSD and were re-engaged to receive a full course of PE as part of the trial. Eligibility criteria included age of 21 or older and a current diagnosis of PTSD as determined by means of the Clinician Administered PTSD Scale for DSM-5 (CAPS-5; Weathers et al., 2018) or a total score above 32 on the PTSD Checklist for DSM-5 (PCL-5; Bovin et al., 2016). Exclusion criteria included active psychosis, dementia, suicidal ideation with clear intent, or concurrent enrollment in another PTSD or depression treatment study. Participants’ ages ranged from 20 to 74 years (mean = 43.9) years and were predominately female (62.7%). Racial/ethnic identity composition of the sample was 47.7% Black, 43.1% White, and 8.3% multiracial, and 7.1% Hispanic. Over half were employed (56.9%), and 59.6% had a VA disability rating. Primary trauma types included military sexual trauma (63.3%), combat (31.2%), and other Criterion A events (5.5%).

### 2.2. Procedure

This study used data from a randomized controlled trial examining whether peer support could enhance re-engagement and completion of PE therapy among veterans with PTSD (Hernandez-Tejada et al., 2024)^1^. Prior approval was obtained from the VAHCS Research and Development Committee and via the institutional review board (IRB) at the university affiliate. Participants were recruited between September 2018 and April 2022 through referrals from VA primary care and outpatient mental health clinics. Eligible veterans completed an intake appointment that included completion of informed consent, diagnostic interviews, and baseline self-report questionnaires.

Following intake, participants were paired with a trained peer and randomly assigned to one of two conditions: (1) PE with peer-assisted in vivo exposure for up to 4 weeks or (2) PE with general peer support for up to 4 weeks. Regardless of conditions, all participants received manualized PE consisting of 12–15 weekly 90-min sessions including psychoeducation, breathing retraining, imaginal exposure to trauma memories, and in vivo exposure to avoided trauma-related cues (Foa et al., 2007).

PTSD symptom levels were assessed at baseline, posttreatment, and at 3- and 6-month follow-ups. Sleep quality was assessed at pre- and post-treatment. Participants received up to USD 140 for completing all study assignments.

### 2.3. Measures

*PTSD Checklist for DSM-5* (PCL-5; Bovin et al., 2016). The PCL-5 is a widely used 20-item self-report measure assessing the presence and severity of PTSD symptoms based on DSM-5 criteria. Items are rated on a 5-point Likert scale ranging from 0 (“Not at all”) to 4 (“Extremely”) for each symptom. Total scores range from 0 to 80. Higher scores reflect greater symptom severity, with a cutoff score of 31–33 demonstrating optimal sensitivity and specificity for probable PTSD on comparison with CAPS-5. The PCL-5 has evidenced good-to-excellent convergent (*r*s = .74 to .85) and discriminant (*r*s = .31 to .60) validity, excellent retest reliability (*r* = .82), and internal consistency (α = .94) (Blevins et al., 2015). Internal consistency in the current study was excellent (α ≥ .92). In line with other studies using the PCL-5 (e.g., Sexton et al., 2017), primary analyses included item 20 that assesses sleep difficulty (*Do you have trouble falling or staying asleep*) because the measure’s psychometric properties are unknown without item 20 and keeping item 20 would allow for comparison between our findings and other sleep-related PTSD literature.

*Pittsburgh Sleep Quality Index* (PSQI; Buysse et al., 1989). The PSQI is a 19-item self-report measure assessing sleep quality and disturbances over the past month in clinical populations. The 19 self-rated items are grouped into seven component scores: subjective sleep quality, sleep latency, sleep duration, habitual sleep efficiency, sleep disturbances, use of sleeping medications, and daytime dysfunction. Daytime dysfunction items index disruption during waking hours due to disturbed sleep (e.g., *during the past month, how often have you had trouble staying awake while driving, eating meals, or engaging in social activity?*). Each of the seven component scores is weighted equally on 0–3 scale, with “0” indicating no difficulty and “3” indicating severe difficulty. The seven component scores are then summed to produce a global PSQI score, which ranges from 0 to 21 points. Higher scores indicate poorer sleep quality. A global score ≥ 5 reflects clinically significant sleep difficulties in at least two areas or moderate difficulties in at least three areas. The PSQI has demonstrated strong internal consistency and test-retest reliability across diverse clinical populations (Beck et al., 2004; Mollayeva et al., 2016; Osorio et al., 2006). PSQI demonstrated modest internal consistency in the current study, with Cronbach’s alpha = .39 at baseline and .66 at post-treatment. These values are somewhat lower than those reported in the original validation study (Buysse et al., 1989) and in other samples (e.g., Cole et al., 2006), though comparable to those observed in certain clinical populations. The PSQI global score reflects multiple, often independent, domains of sleep, which can reduce internal consistency, particularly in heterogeneous samples or during treatment when specific sleep domains may improve at different rates (Mollayeva et al., 2016). Thus, lower internal consistency may reflect the multidimensional structure of the PSQI rather than poor measurement quality.

### 2.4. Statistical Analysis

Data from each phase of the study were screened for missingness (see Table 1 for completion rates). PSQI data were visually screened for extreme values caused by entry errors (e.g., entering standard time instead of military time) and manually corrected. Analyses were conducted in IBM SPSS Statistics (Version 22; Armonk, NY, USA)and SAS (Version 9.4, Cary, NC, USA). To characterize the data, we calculated bivariate correlations between PTSD and sleep variables. For primary analyses, change in sleep indices (measured by the PSQI total score and subscales) and PTSD symptom levels (measured by the PCL-5) were operationalized as residualized change scores from pre- to post-treatment. These were calculated by outputting the residuals of a regression model where the pre-treatment score predicted the post-treatment score. Lower residualized change scores reflect greater improvement adjusted for pre-treatment levels.

First, Pearson correlations were calculated among change scores to examine concurrent associations between changes in sleep and changes in PTSD symptoms from pre- to post-treatment. Next, preliminary hierarchical linear models (HLMs) estimated in SAS PROC MIXED were used to examine whether changes in sleep (PSQI global and seven subscale scores) predicted PTSD symptom severity (PCL-5) across time, encompassing post-treatment and 3- and 6-month follow-up PCL-5 scores. Models were estimated using restricted maximum likelihood and included random intercepts and slopes to account for individual variability in intercepts and slopes over time, with an unstructured covariance structure. Time was coded such that the intercept reflected scores at post-treatment. Full models included covariates for age, sex, treatment condition (and its interaction with time), and baseline PTSD severity (and its interaction with time). If the interaction between time and sleep change was non-significant, it was removed to examine the main effect of sleep change on post-treatment PTSD symptom severity. We considered *p*-values less than .05 significant and less than .08 trends. To assess the robustness of our findings against multiple comparisons, we applied the Benjamini–Hochberg false discovery rate (FDR) correction across the 16 *p*-values (8 main effects and 8 interaction effects). In addition, follow-up analyses were conducted using a PCL-5 score with the sleep item removed. Models make use of all available data consistent with an intent-to-treat approach.

## 3. Results

Descriptive statistics and bivariate correlations between key variables are reported in Table 1.

### 3.1. Concurrent Associations Between Sleep Change and PTSD Symptom Change

Pearson correlations between residualized PSQI scores and residualized PCL-5 scores are reported in Table 2. Greater improvements in global sleep quality (*r* = .34, *p* = .009), subjective sleep quality (*r* = .30, *p* = .021), and daytime dysfunction (*r* = .41, *p* = .001) were significantly associated with greater reductions in PTSD symptoms. Sleep disturbance showed a marginal association with greater reductions in PTSD symptoms at trend-level significance (*r* = .25, *p* = .056). In contrast, changes in sleep latency, duration, efficiency, and medication use were not significantly correlated with PTSD symptom change. The pattern of results was consistent using the modified PCL-5 score, excluding the sleep item.

### 3.2. Sleep Change as a Predictor of PTSD Symptoms over Time

Results for the eight full models (including covariates) are summarized in Table 3, with a focus on the primary tests of interest: the main effects of sleep change on post-treatment PTSD severity and on the slope of PTSD change across follow-up. Detailed results tables are included in the supplement, including for preliminary models (without covariates). Overall, inclusion of covariates did not change the pattern of results. Across all models, there were no significant effects for age, sex, or treatment condition on outcome. In each model, baseline PTSD severity significantly predicted higher post-treatment PTSD severity. However, the interaction of baseline PTSD severity with time was non-significant; baseline PTSD severity did not predict slopes of PTSD symptoms across follow-up.

Greater improvement in global sleep quality significantly predicted lower PTSD symptom severity at post-treatment, *b* = 1.83, *SE* = .80, *t*(47) = 2.29, *p* = .03, but did not predict the PTSD symptoms across follow-up (*p* = .80). A trend-level effect was found between improvements in subjective sleep quality and lower PTSD symptoms at post-treatment, *b* = 6.64, *SE* = 3.80, *t*(45) = 1.75, *p* = .09; but not across follow-up (*p* = .99). Similarly, a trend-level effect was found between improvements in sleep disturbance and lower PTSD symptoms at post-treatment, *b* = 8.40, *SE* = 4.52, *t*(46) = 1.86, *p* = .070; but not across follow-up (*p* = .69). Greater improvement in daytime dysfunction predicted lower PTSD symptoms at post-treatment, *b* = 8.52, *SE* = 2.67, *t*(45) = 3.19, *p* = .003. A significant time interaction indicated that greater improvement in daytime dysfunction predicted PTSD symptom *worsening* across follow-up, *b* = −1.99, *SE* = 0.94, *t*(45) = −2.11, *p* = .04^2^. Finally, sleep latency, sleep duration, habitual sleep efficiency, and medication use^3^, were not significant predictors of PTSD symptom intercepts or slopes (all *p*s > .08).

A robustness check using PCL scores with the sleep-related item removed yielded the same pattern of results, indicating that observed effects were not attributable to construct overlap between measures. Additionally, we applied a Benjamini–Hochberg FDR correction to the 16 post-treatment tests. After correction, only the association between improvement in daytime dysfunction and lower PTSD symptoms at post-treatment remained statistically significant.

## 4. Discussion

The current study examined the relationship between changes in sleep quality and posttraumatic stress symptoms during and following PE. Two complementary analytic approaches revealed evidence for an association between sleep and PTSD symptom change during treatment. First, residualized change scores showed that greater improvements in global sleep quality were significantly correlated with greater reductions in PTSD symptom severity from pre- to post-treatment. This finding is consistent with previous research identifying co-occurring improvements in overall sleep quality and PTSD symptoms across the course of trauma-focused treatment (e.g., Sherrill et al., 2022; Taylor et al., 2020). Furthermore, contrary to our hypothesis, improvements in subjective sleep quality and daytime dysfunction, but not total sleep time, sleep efficiency, and sleep disruptions, were significantly linked to greater reductions in PTSD symptom severity pre- to post-treatment, suggesting that these sleep components may be more important to PE outcomes. Second, results from a hierarchical linear model demonstrated that greater improvement in global sleep quality predicted lower PTSD symptom severity at post-treatment, even when controlling for baseline PTSD symptoms. However, this relationship did not extend to follow-up assessments at 3 and 6 months, suggesting that the beneficial effects of overall sleep quality improvement may be limited to the period of active treatment. Although these findings partially align with theoretical accounts proposing a role for sleep in emotional processing and therapeutic learning during treatment (e.g., Kleim et al., 2016; Pace-Schott et al., 2015), it is noteworthy that the sleep domain most consistently associated with PE outcomes in this study—particularly daytime dysfunction—does not directly reflect the nighttime sleep processes emphasized in most mechanistic models.

### 4.1. Unexpected Role of Daytime Dysfunction

The sleep domain most strongly and consistently associated with treatment outcomes was not nighttime processes such as sleep disturbances, sleep efficiency, or total sleep time, but rather daytime dysfunction—a measure of how sleep affects alertness and performance during waking hours. The PSQI Daytime Dysfunction subscale includes two items: difficulty staying awake during daytime activities (e.g., eating, driving, or socializing), and trouble maintaining enthusiasm to complete tasks. Improvements in daytime dysfunction were associated with concurrent reductions in symptom levels and lower PTSD symptom severity at post-treatment. One possible explanation is that impaired attentional engagement due to poor sleep (Lim & Dinges, 2010) may interfere with the initial encoding of safety learning during PE (Cowan, 1988; Robinson, 1995). Before information can be consolidated during sleep, it must first be effectively encoded during exposure exercises during the day. Daytime alertness may be critical for attending to, emotionally engaging with, and processing the corrective learning that occurs during imaginal and in vivo exposures (Ericsson & Kintsch, 1995; Schweppe & Rummer, 2014; Unsworth, 2016; Woodman & Chun, 2006). From this perspective, individuals who experience substantial daytime impairment may be less able to fully engage during exposure exercises, whereas those whose daytime functioning improves, irrespective of sleep metrics per se, may be better able to attend to the outcomes of exposure events. This may explain why several nighttime sleep metrics related to memory consolidation (i.e., sleep disturbance, total sleep time, sleep efficiency) did not significantly predict PE outcomes.

Alternatively, the observed association between improvements in daytime dysfunction and PTSD symptom levels may reflect the reverse direction—that is, as PTSD symptoms improve during treatment, individuals may experience greater daytime functioning independent of changes in sleep per se, particularly given that the majority of PTSD symptoms occur during waking hours. Taken together, these findings suggest that poor sleep quality—particularly its impact on daytime functioning—may interfere with PE by degrading learning processes presented by exposure during the day rather than at night. Daytime dysfunction may serve as a critical bridge linking sleep quality, attentional bias, and PTSD treatment outcomes.

Nevertheless, greater improvements in daytime dysfunction during PE predicted *increases* in symptom severity across follow-up. One possibility is that this reflects a form of regression to the mean. Participants who experienced the greatest improvements during treatment—both in daytime functioning and PTSD symptoms—also may have had greater potential for subsequent regression toward their individual mean levels of functioning. Indeed, the average increase in PTSD symptoms from posttreatment to 6-month follow-up (approximately 3.4 points on the PCL-5) was small relative to the overall symptom reduction during treatment (~19-point drop), suggesting that even modest symptom reemergence may appear more pronounced in those who initially benefited the most. This pattern aligns with findings from studies indicating that while PE generally leads to sustained symptom reduction, some individuals experience slight increases in PTSD symptoms during follow-up periods (e.g., Klaeth et al., 2024; Thorp et al., 2019).

### 4.2. Clinical Implications

Several studies have found that sleep problems do not predict PE outcomes (Sexton et al., 2017; Koffel et al., 2016; Galovski et al., 2016), suggesting that addressing sleep within the context of PE may not be necessary to enhance treatment efficacy. In contrast, other work suggests that poor sleep may contribute to poorer response to trauma-focused therapy (López et al., 2017; Pruiksma et al., 2023; Straus et al., 2015; Taylor et al., 2020; Zayfert & DeViva, 2004). The present study offers evidence in favor of the latter view, indicating that improvements in sleep quality, particularly subjective sleep quality and daytime dysfunction, during treatment may facilitate therapeutic gains, even when PTSD symptoms are already improving. These results support the notion that adjunctive sleep interventions—whether prior to or alongside PE—could enhance the effectiveness of treatment, especially for patients presenting with pronounced daytime impairment. Consistent with this, prior studies show superior PE outcomes when cognitive behavioral therapy for insomnia (CBT-I) was delivered concurrently (Walters et al., 2020; Colvonen et al., 2019) or sequentially (Baddeley & Gros, 2013). Similarly, veterans with obstructive sleep apnea (OSA) experienced greater benefit from CPT when using continuous positive airway pressure (CPAP) therapy concurrently (Mesa et al., 2017).

Although nighttime sleep processes have received the bulk of empirical and theoretical attention in PTSD research (Germain et al., 2005; Pace-Schott et al., 2015), the present findings highlight the clinical relevance of daytime dysfunction—a dimension that reflects the impact of poor sleep on daytime alertness and cognitive functioning (Buysse et al., 1989; Taylor & Pruiksma, 2014). One potential clinical implication of these findings concerns the cognitive processing phase of PE, during which individuals reflect on what they learned from imaginal and in vivo exposures, evaluate how their expectations were confirmed or disconfirmed, and articulate revised beliefs about safety and threat (Foa et al., 2007). Individuals who experience daytime fatigue due to poor sleep may struggle to fully attend to or integrate these corrective experiences (Altena et al., 2008; Lim & Dinges, 2010) and may benefit from additional support during cognitive processing. For example, clinicians could prioritize helping individuals identify and elaborate on core lessons, such as recognizing that trauma memories and trauma-related cues are not dangerous (Foa et al., 2007). Monitoring alertness and capacity to attend to exposure exercises during treatment may help identify those who could benefit from these targeted supports to optimize therapeutic learning.

### 4.3. Limitations

Several limitations of the current study warrant consideration. First, the PSQI is a subjective measure of sleep quality and is subject to biases related to retrospective recall and subjective interpretation (Buysse et al., 2006); however, it is arguably the most direct indicator of perceived sleep disturbance—a factor closely linked to distress and functional impairment in PTSD (Zayfert & DeViva, 2004; Germain, 2013). Furthermore, the poor internal consistency of the PSQI global score, particularly at baseline, is lower than typically reported. This significantly limits the interpretability of findings that rely on the global score. Poor internal consistency of the PSQI is not without precedent in veteran or clinical populations (e.g., Cole et al., 2006; Matsangas & Mysliwiec, 2018) and may reflect the PSQI’s multidimensional structure and its reduced sensitivity to specific sleep disorders common in veterans, such as obstructive sleep apnea (Mysliwiec et al., 2013) or service-related sleep disturbances (Insana et al., 2013). Future research could incorporate the PSQI Addendum for PTSD (Germain et al., 2005), which was specifically developed to assess trauma-related sleep disturbances in military populations.

Second, the associations between residualized change scores in sleep and PTSD symptoms preclude any directionality. It remains unclear whether sleep improvements drive symptom reduction, whether symptom relief improves sleep, or whether PE influences both during treatment. More frequent assessments—ideally session-by-session or daily—could clarify temporal sequencing (Sexton et al., 2017). For example, Hunt et al. (2023) used nightly sleep diaries and weekly PTSD assessments to show that improvements in sleep efficiency preceded greater fear extinction during PE, offering one possible pathway by which sleep may influence treatment response. However, no study to our knowledge has taken this approach to examine the role of daytime dysfunction, highlighting an important direction for future research, particularly in light of our findings. Furthermore, we did not administer the PSQI at follow-up; future studies would benefit from continuing to measure sleep during treatment follow-up to evaluate how PTSD symptoms *and* sleep may continue to change following PTSD treatment. That said, a strength of the current study is the use of prospective models to evaluate change in sleep as a predictor of PTSD symptoms following treatment.

Third, PE was delivered in a non-standard way (i.e., with peer support) to veterans, who experience higher rates of risk factors for poor sleep, including sleep apnea and chronic pain, than civilians (Mysliwiec et al., 2013; Kerns et al., 2003). Thus, findings may not generalize beyond veterans,

Finally, only one effect—daytime dysfunction predicting post-treatment PTSD severity—remained significant after we applied a Benjamini-Hochberg FDR correction. Although this strengthens confidence in that finding, it also underscores the need for replication and cautious interpretation of trend-level effects.

### 4.4. Conclusions

The present study underscores the role of sleep quality, particularly daytime functioning, in shaping PTSD symptom outcomes during PE. Improvements in global and subjective sleep quality were associated with greater symptom reductions during treatment, with global sleep predicting lower PTSD severity at post-treatment. Improvements in daytime dysfunction showed a similar pattern—predicting both greater symptom reduction during treatment and lower post-treatment severity—but were also unexpectedly linked to symptom worsening at follow-up. These findings suggest that how sleep affects daytime alertness and cognitive functioning may matter more for treatment response than specific disruptions to nighttime sleep processes.

## Figures and Tables

**Table 1:** Descriptive Statistics and Bivariate Correlations.

	n	Mean	SD	1	2	3	4
1. Baseline PCL-5	108	54.74	14.56	—			
2. Post-PCL-5	75	35.24	20.86	.49 **	—		
3. 3 Month PCL-5	62	35.79	22.07	.41 **	.83 **	—	
4. 6 Month PCL-5	64	38.70	21.39	.52 **	.82 **	.92 **	—
**Baseline PSQI**							
5. Subjective Sleep Quality	107	2.25	0.67	.32 **	.31 **	.36 **	.40 **
6. Sleep Latency	87	2.44	0.87	.11	−.04	.06	.03
7. Sleep Duration	92	2.41	0.83	−.00	.19	.43 **	.33*
8. Sleep Efficiency	90	1.71	1.23	.02	.02	.06	.02
9. Sleep Disturbance	108	2.17	0.63	.33 **	.17	.16	.13
10. Sleep Medication	109	1.92	1.23	.15	.10	−.10	−.01
11. Daytime Dysfunction	109	1.93	0.87	.42 **	.30 **	.30 *	.34 **
12. Global Score	109	11.68	3.38	.23*	.19	.17	.11
**Post-treatment PSQI**							
13. Subjective Sleep Quality	60	2.05	0.87	.35 **	.51 **	.47 **	.65 **
14. Sleep Latency	53	2.25	0.96	.10	.19	.22	.23
15. Sleep Duration	57	2.04	0.98	.07	.28 *	.30 *	.33 *
16. Sleep Efficiency	56	1.55	1.21	.05	.01	.05	.08
17. Sleep Disturbance	61	1.74	0.73	.56 **	.47 **	.40 **	.47 **
18. Sleep Medication	60	1.9	1.20	.30*	.17	.17	.18
19. Daytime Dysfunction	60	1.53	0.98	.31 *	.51 **	.47 **	.44 **
20. Global Score	61	10.54	3.64	.28 *	.45 **	.37 **	.48 **

Note. PCL-5 = PTSD Checklist of DSM-5. PSQI = Pittsburgh Sleep Quality Index. Means and standard deviations reflect untransformed scores. * *p* < .05. ** *p* < .01.

**Table 2:** Bivariate Correlations Among Residualized PCL-5 and PSQI Scores.

	1	2	3	4	5	6	7	8	9
1. PCL-5	—								
2. Subjective Sleep Quality	.30 *	—							
3. Sleep Latency	.14	.20	—						
4. Sleep Duration	.18	.19	.15	—					
5. Sleep Efficiency	−.05	−.06	.23	.49 **	—				
6. Sleep Disturbance	.25	.40 **	.34 *	.00	.07	—			
7. Sleep Medication	.04	.01	.02	−.04	.23	−.14	—		
8. Daytime Dysfunction	.41 **	.41 **	.1	.09	.02	.20	.03	—	
9. Global Score	.34 **	.50 **	.61 **	.57 **	.62 **	.36 **	.23	.44 **	—

Note. PCL-5 = PTSD Checklist of DSM-5. PSQI = Pittsburgh Sleep Quality Index. Variables are residualized change scores from pre- to post-treatment. * *p* < .05. ** *p* < .01.

**Table 3:** Hierarchical Linear Model Estimates of Sleep Change Predicting PTSD Symptoms.

Change in PSQI Domain (n)	Post-Treatment PCL-5 (Intercept)					PCL-5 Across Time (Slope)				
	*b*	*SE*	*t*	*p*	*q*	*b*	*SE*	*t*	*p*	*q*
Subjective Sleep Quality (58)	6.64	3.8	1.75	.09	.24	0.00	1.35	0.00	.99	1.00
Sleep Latency (43)	2.17	3.42	0.64	.53	.72	0.63	1.20	0.53	.60	.74
Sleep Duration (49)	2.78	3.87	0.72	.48	.72	−0.85	1.32	−0.65	.52	.72
Sleep Efficiency (47)	−1.64	2.60	−0.63	.53	.72	0.73	0.88	0.82	.41	.72
Sleep Disturbance (59)	8.40	4.52	1.86	.07	.24	−0.65	1.61	−0.41	.69	.79
Medication Use (59)	1.54	2.49	0.62	.54	.72	1.47	0.82	1.79	.08	.24
Daytime Dysfunction (59)	8.52	2.67	3.19	<.01	.04	−1.99	0.94	−2.11	.04	.21
Global Sleep Quality (60)	1.83	0.80	2.29	.03	.21	−0.07	0.27	−0.26	.80	.85

Note. PCL-5 = PTSD Checklist of DSM-5. PSQI = Pittsburgh Sleep Quality Index. *q* = Benjamini–Hochberg adjusted *p*-values. PSQI variables are residualized change scores from pre- to post-treatment. The table reports key estimates from eight models examining how changes in each sleep domain predict PCL-5 intercepts at post-treatment and slopes across follow-up. As summarized in the text, all models controlled for age, sex, treatment condition (and its interaction with time), and baseline PTSD severity (and its interaction with time).

## Data Availability

Data supporting the findings of this study are available from the authors upon reasonable request and with the permission of the Veterans Affairs Medical Center of Charleston, South Carolina.

## Notes

1 Peer-support condition from the parent study had no significant impact on the current study’s findings.

2 This effect only became significant after controlling for baseline PCL in the model (see Table S7 in Supplementary Materials).

3 Medication use predicted PTSD symptom slope at a trend level, *p* = .08, but this was not replicated in the robustness check.

## Supplementary Materials

The following supporting information can be downloaded at: https://www.mdpi.com/article/10.3390/bs15121654/s1.
