# Supplementary material for: Differential Associations Between Sleep Domains and Response to Prolonged Exposure Therapy
Source: Behav Sci (Basel). 2025 Dec 2;15(12):1654. doi: 10.3390/bs15121654 (PMC12729736; doi:10.3390/bs15121654)
Supplement: Supplementary file 1 [file behavsci-15-01654-s001.zip › behavsci-3926252-supplementary.pdf]

Supplemental Materials

Table S1

Estimates of Subjective Sleep Quality Predicting PTSD Symptoms

	b	*SE*	*DF*	*t*	*p*
Preliminary Model					
Intercept	34.56	2.65	57	13.06	<.0001
Time	1.58	0.84	55	1.89	.064
Subjective Δ	10.39	3.69	46	2.82	.007
Time * Subjective Δ	0.35	1.20	46	0.29	.772
Full Model					
Intercept	-9.31	13.75	52	-0.68	.501
Age	0.19	0.21	45	0.9	.371
Sex	0.34	4.99	45	0.07	.947
Time	-0.21	3.43	52	-0.06	.951
Condition	0.31	4.97	45	0.06	.951
Time * Condition	0.88	1.74	45	0.51	.616
Baseline PCL	0.63	0.17	45	3.73	.001
Time * Baseline PCL	0.03	0.06	45	0.43	.669
Subjective Δ	6.64	3.80	45	1.75	.088
Time * Subjective Δ	0.001	1.35	45	0.00	.999
Main Effect, with Interaction Removed					
Subjective Δ	6.65	3.66	45	1.82	.076

Table S2

Estimates of Sleep Latency Predicting PTSD Symptoms

	b	***SE***	***DF***	***t***	***p***
Preliminary Model					
Intercept	34.43	3.32	42	10.36	<.0001
Time	0.93	0.97	40	0.96	.341
Latency Δ	4.76	3.96	33	1.2	.238
Time * Latency Δ	0.89	1.13	33	0.78	.439
Full Model					
Intercept	-22.79	14.68	37	-1.55	.129
Age	0.29	0.22	32	1.35	.188
Sex	2.96	5.85	32	0.51	.616
Time	1.93	3.73	37	0.52	.609
Condition	4.50	5.80	32	0.78	.443
Time * Condition	1.84	2.05	32	0.89	.378
Baseline PCL	0.76	0.19	32	4.07	.000
Time * Baseline PCL	-0.03	0.06	32	-0.53	.598
Latency Δ	2.17	3.42	32	0.64	.530
Time * Latency Δ	0.63	1.20	32	0.53	.601
Main Effect, with Interaction Removed					
Latency Δ	2.84	3.18	32	0.89	.379

Table S3

Estimates of Sleep Duration Predicting PTSD Symptoms

	b	***SE***	***DF***	***t***	***p***
Preliminary Model					
Intercept	33.21	3.08	48	10.79	<.0001
Time	1.01	0.97	46	1.04	.303
Duration Δ	3.45	3.84	38	0.9	.374
Time * Duration Δ	-0.70	1.25	38	-0.56	.579
Full Model					
Intercept	-9.42	14.89	43	-0.63	.531
Age	0.22	0.24	37	0.91	.368
Sex	-2.24	5.94	37	-0.38	.708
Time	-1.10	3.65	43	-0.3	.765
Condition	-0.09	5.84	37	-0.02	.988
Time * Condition	2.27	2.02	37	1.12	.269
Baseline PCL	0.63	0.19	37	3.36	.002
Time * Baseline PCL	0.02	0.06	37	0.28	.779
Duration Δ	2.78	3.87	37	0.72	.477
Time * Duration Δ	-0.85	1.32	37	-0.65	.522
Main Effect, with Interaction Removed					
Duration Δ	2.13	3.73	37	0.57	.572

Table S4

Estimates of Sleep Efficiency Predicting PTSD Symptoms

	b	***SE***	***DF***	***t***	***p***
Preliminary Model					
Intercept	32.78	3.19	46	10.29	<.0001
Time	1.03	0.99	44	1.04	.302
Efficiency Δ	-0.88	2.72	37	-0.32	.748
Time * Efficiency Δ	0.83	0.85	37	0.98	.336
Full Model					
Intercept	-15.30	14.70	41	-1.04	.304
Age	0.33	0.24	36	1.35	.186
Sex	-2.97	6.03	36	-0.49	.625
Time	-0.79	3.66	41	-0.21	.831
Condition	2.78	5.91	36	0.47	.641
Time * Condition	1.75	2.07	36	0.84	.404
Baseline PCL	0.61	0.19	36	3.27	.002
Time * Baseline PCL	0.02	0.06	36	0.28	.782
Efficiency Δ	-1.64	2.60	36	-0.63	.532
Time * Efficiency Δ	0.73	0.88	36	0.82	.415
Main Effect, with Interaction Removed					
Efficiency Δ	-1.07	2.51	36	-0.43	.673

Table S5

Estimates of Sleep Disturbance Predicting PTSD Symptoms

	b	***SE***	***DF***	***t***	***p***
Preliminary Model					
Intercept	34.60	2.51	58	13.8	<.0001
Time	1.30	0.86	56	1.52	.134
Disturbance Δ	14.27	3.94	47	3.62	.001
Time * Disturbance Δ	0.30	1.35	47	0.22	.825
Full Model					
Intercept	-1.48	14.19	53	-0.1	.917
Age	0.19	0.21	46	0.93	.356
Sex	-2.54	4.94	46	-0.51	.609
Time	-2.34	3.62	53	-0.65	.520
Condition	-1.67	4.89	46	-0.34	.733
Time * Condition	1.31	1.75	46	0.75	.458
Baseline PCL	0.53	0.18	46	2.99	.004
Time * Baseline PCL	0.06	0.06	46	0.88	.383
Disturbance Δ	8.40	4.52	46	1.86	.070
Time * Disturbance Δ	-0.65	1.61	46	-0.41	.687
Main Effect, with Interaction Removed					
Disturbance Δ	7.88	4.34	46	1.82	.076

Table S6

Estimates of Sleep Medication Predicting PTSD Symptoms

	b	***SE***	***DF***	***t***	***p***
Preliminary Model					
Intercept	35.03	2.72	58	12.87	<.0001
Time	1.13	0.83	56	1.36	.178
Medication Δ	2.73	2.73	47	1	.321
Time * Medication Δ	1.47	0.81	47	1.82	.074
Full Model					
Intercept	-9.94	13.19	54	-0.75	.454
Age	0.25	0.21	45	1.19	.240
Sex	-1.39	4.87	45	-0.29	.776
Time	-1.50	3.18	53	-0.47	.638
Condition	-0.69	4.95	45	-0.14	.890
Time * Condition	1.45	1.70	45	0.86	.396
Baseline PCL	0.64	0.17	45	3.88	.000
Time * Baseline PCL	0.04	0.06	45	0.66	.513
Medication Δ	1.54	2.49	45	0.62	.540
Time * Medication Δ	1.47	0.82	45	1.79	.080
Main Effect, with Interaction Removed					
Medication Δ	2.95	2.36	45	1.25	.218

Table S7

Estimates of Daytime Dysfunction Predicting PTSD Symptoms

	b	***SE***	***DF***	***t***	***p***
Preliminary Model					
Intercept	35.02	2.45	58	14.29	<.0001
Time	1.12	0.84	56	1.33	.189
Dysfunction Δ	10.52	2.74	47	3.84	.0004
Time * Dysfunction Δ	-1.47	0.92	47	-1.6	.116
Full Model					
Intercept	-2.74	12.61	54	-0.22	.829
Age	0.21	0.20	45	1.09	.283
Sex	-3.62	4.68	45	-0.78	.442
Time	-3.67	3.22	53	-1.14	.260
Condition	-2.61	4.59	45	-0.57	.573
Time * Condition	1.66	1.70	45	0.98	.334
Baseline PCL	0.57	0.15	45	3.72	.001
Time * Baseline PCL	0.07	0.06	45	1.32	.192
Dysfunction Δ	8.52	2.67	45	3.19	.003
Time * Dysfunction Δ	-1.99	0.94	45	-2.11	.040

Table S8

Estimates of Global Sleep Quality Predicting PTSD Symptoms

	b	***SE***	***DF***	***t***	***p***
Preliminary Model					
Intercept	34.63	2.52	59	13.77	<.0001
Time	1.21	0.85	57	1.43	.158
Global Δ	2.52	0.78	48	3.23	.002
Time * Global Δ	0.03	0.26	48	0.12	.902
Full Model					
Intercept	0.76	13.72	54	0.06	.956
Age	0.10	0.21	47	0.47	.641
Sex	-1.64	4.70	47	-0.35	.728
Time	-2.29	3.32	54	-0.69	.494
Condition	-3.24	4.81	47	-0.67	.505
Time * Condition	1.51	1.74	47	0.87	.389
Baseline PCL	0.58	0.16	47	3.61	.001
Time * Baseline PCL	0.05	0.06	47	0.89	.379
Global Δ	1.83	0.80	47	2.29	.027
Time * Global Δ	-0.07	0.27	47	-0.26	.795
Main Effect, with Interaction Removed					
Global Δ	1.77	0.76	47	2.32	.025
